# How Effective is Fibula Pro Tibia Plating in Treatment of Distal Tibial Fractures: A Pilot Study

**DOI:** 10.5704/MOJ.2407.004

**Published:** 2024-07

**Authors:** S Jain, H Khare, K Verma, U Kumar, A Ajmera

**Affiliations:** Department of Orthopaedics, Mahatma Gandhi Memorial Government Medical College, Indore, India

**Keywords:** distal tibia fracture, plafond fracture, fibula pro tibia

## Abstract

**Introduction:**

Despite recent advances, management of distal tibial fractures is challenging, with high rate of complications. Fibula pro tibia plating technique fixes fibula and tibia together, via laterally placed fibular plate without disturbing the tibial soft tissue sleeve. We contemplated this pilot study to assess effectiveness of fibula pro tibia plating in management of distal tibia fibula fractures.

**Materials and Methods:**

A total of 30 patients with distal tibia fibula fractures with fracture line extending within 5cm from tibial plafond were managed with fibula pro tibia plating, with or without minimal articular fixation. Outcome evaluation was done by union, union time, alignment and functional outcome as assessed by AOFAS score.

**Results:**

Mean age in the series was 39.4 years with male to female ratio of 3:2. Mean duration of surgery, blood loss and C arm exposure were 79 minutes (range 52 to 98min), 80ml (range 62 to 102ml) and 48 shoots (range 36 to 81 shoots), respectively. All fractures united in mean union time of 10.2 weeks (range 9 to 14 weeks) with acceptable alignment in all the patients except one. Mean AOFAS score was 86.3 (range 70 to 93) with 29 patients having good to excellent outcome. One patient had varus malunion and in one case infection was seen.

**Conclusion:**

Fibula pro tibia plating can be successfully used to manage complex distal tibia fractures which leaves the soft tissue and periosteal sleeve undisturbed, thus avoiding wound related problems and leading to early union.

## Introduction

Tibial fractures in the lower part of leg include fractures of distal tibia and plafond fractures. Distal tibia fractures are particularly extra-articular fractures located between 4 to 10cm from the tibial articular surface, whereas plafond fractures are defined as injuries that involve the articular weight-bearing surface of the distal tibia equating the distal tibial articular surface as the ceiling of the ankle joint^1,2^. Both these fractures distal tibial and pilon fractures are generally (75 to 90%) associated with fibula fracture^3^. This is because of complex associated anatomy and the transmission of forces along the interosseous membrane to fibula causing fracture of both tibia and fibula. Associated fracture of the fibula increases comminution and severity of these tibial fractures, thus affecting their management^4^.

Management of these distal tibial fractures with or without articular involvement is always a challenge to treat with options ranging from conservative with cast, open reduction and internal fixation, external fixation with or without limited internal fixation, intramedullary nailing to minimal invasive plate osteosynthesis^5-7^. Despite recent advances and progress in surgical procedures, reported rate of complications associated with these fractures is 20 to 56%^8,9^. Reasons for this high rate of associated complications making surgical intervention hazardous are many. Firstly, distal part of the tibia is subcutaneous and there is insufficiency of soft tissue coverage and relatively tenuous blood supply in this part of tibia resulting in poor healing potential^9^. Secondly, these fractures are usually result of high energy trauma which results in severe surrounding soft tissue trauma, substantial articular impaction, associated multiple injuries, which may require intensive care and makes the fracture pattern multi-fragmentary^10^. Hence due to the fact, that the composite complex tibia-fibular anatomy around ankle, poor soft tissue coverage, high energy trauma, complex fracture geometry, substantial comminution and articular involvement, compromised blood flow, lack of ideal implant availability, small bone stock to hold implant and limited opportunities for surgical incision/approach, management of these fractures are associated with high rate of complications like wound dehiscence, infection, nonunion, malunion and post traumatic arthritis etc.

The key element in preventing these complications is minimal disturbance of the soft tissue envelope, avoiding extensive periosteal stripping and maintenance of the vascularity of the soft tissue sleeve to provide perfusion to the myocutaneous tissue and underlying bone needed for healing^11,12^. Fibula pro tibia plating is a type of construct made by extending the screws from the fibular plate to the tibia passing across tibiofibular space^13^. Thus, fibula pro tibia screws do not touch or disturb the medial tibial soft tissue envelope and retains the integrity of periosteal sleeve and vascularity. In this pilot study, we evaluated the outcome of this fibula pro tibia plating technique in management of distal tibial and pilon fractures.

## Materials and Methods

This prospective interventional study was done in 30 patients with distal tibia and fibula fractures presented to our centre from 2019 to 2021, which were managed using fibula pro tibia plating, with or without minimal articular fixation. Ethical and scientific approval from the institutional review board was taken prior to initiation of the study. All patients between 18 to 60 years with distal tibia fibula fractures with tibial fracture line extending within 5cm from the tibial plafond with or without the involvement of articular surface were included in the study. Only patients operated within 7 days of injury were included in the study. Patients having open fibular fractures of Gustilo Anderson grade 3B and above were excluded from the study as these were not amenable to fibular plate fixation. Patients with open tibial fractures i.e. wound over medial or anterior part of leg, were included in the study. Patients with pathological fractures and ipsilateral fractures of same limb were excluded from the study. Well informed written consent was taken from all the patients.

All patients were evaluated with proper history, clinical examination and standard AP and lateral radiographs. All patients were initially haemodynamically stabilised along with wound lavage, IV fluids, antibiotics, and analgesics. After obtaining fitness for surgery all patients were operated under spinal anaesthesia in supine position with sandbag under the buttock of affected side. Surgery was done under tourniquet control and with fluoroscopic guidance.

**Fig. 1:**
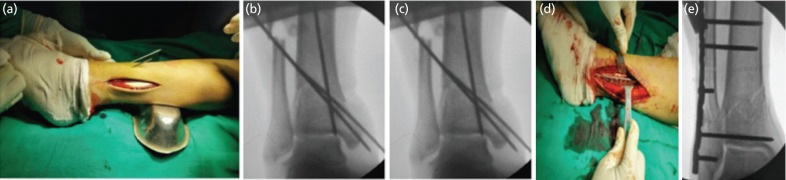
(a, b, c) Intra-operative photograph and fluoroscopic views of fibula pro tibia plating showing fibular exposure, reduction, and provisional fixation with K wire and (d, e) fixation with fibula pro tibia screws plate.

A standard lateral incision was used to expose the fibula. Laterally exposed fibular fracture was open reduced with the help of bone holders. A locking fibular plate was applied to the posterolateral surface of the fibula and provisionally fixed with k wires in proximal and distal segments. After open reduction of fibula and provisional plate application, gentle traction along with necessary rotation was done and closed reduction of the tibial fracture was obtained. Close reduction of the tibia so obtained was checked and confirmed in both AP and lateral views on C arm and provisionally fixed by passing 2 or 3 k wires (2.5mm) percutaneously. Percutaneously, cannulated cancellous screws or additional k wires were passed to hold the articular reduction if needed. Acceptable reduction and alignment of both tibia and fibula fracture was re-confirmed under C-arm in both the views. Fibula pro tibia screws were inserted through the provisionally fixed fibular plate, with direction 250 to 300 directed anteriorly, extending laterally from the plate, to fibula, the tibio-fibular space and finally into the medial tibial cortex. Anterior and medial tibial periosteal sleeve was left undisturbed. Before putting the fibula pro tibia screws the ankle was held in maximum attainable dorsi-flexion, so as not to narrow the ankle mortise. Proximal to the fracture line was the first screw which was inserted, and it was a non-locking screw. Subsequently other fibula pro tibia locking screws were passed both proximally and distally to the tibial fracture line, with at least three screws, proximal to fracture line. Provisional k wires used for fixation were removed and thorough lavage of the wound was done. Lateral surgical incision was closed in layers followed by application of a below knee slab (Fig. 1).

Post-operatively, patients were given IV antibiotics and analgesics for 3 to 5 days. Non weight bearing walker assisted ambulation and knee range of motion exercises were started from post-operative day one. Sutures and below knee slab removal was done at two weeks. Following slab removal ankle mobilisation and toe touch weight bearing were started. Gradually partial weight bearing was started, and full weight bearing was delayed until union was seen on plain radiographs in three of the four cortices in two views. Patients were followed for minimum of 12 months.

Outcome assessment was done by intra-operative parameters and by radiological and functional criterion. All patients included in the study were assessed for intra-operative parameters of duration of surgery, blood loss and C arm exposure. At final follow-up, radiological analysis was done on antero-posterior plain radiograph for union status and alignment. Time taken for union and alignment on radiographs was noted. Functional outcome was assessed as per AOFAS score (100 points) and the patients were graded accordingly as excellent (86-100), good (71-85), fair (51-70) and poor (<50).

## Results

A total of 30 patients of distal tibial fractures with mean age 39.4 years (range 20 to 55 years) were included in the study. Eighteen were male and 12 patients were female. Sixteen patients sustained fracture of right side whereas 14 sustained left side fracture. All the fractures were result of high energy trauma, with 26 sustained due to road traffic accident and 4 due to fall from height. As per the OTA classification, 18 were type A and 12 were type C. Eight fractures were closed fractures, 6 were open grade 1, 10 had grade 2 and 6 were open grade 3A as per Gustilo Anderson grade. The mean delay in surgery was 4.13 days (range 1 to 7 days). Mean follow-up was 14.4 months (range 12 to 23 months) (Table 1).

**Table 1:** Results of 30 patients who underwent fibula pro tibia plating for distal tibial fractures.

Parameter	Mean + SD (range)
Age	39.4 + 10.76 years (20 to 55)
Follow-up	14.4 months (12 to 23)
Injury to surgery time	4.13 + 1.4 days (1 to 7)
Intra-operative parameters	
a) Duration of surgery	79 + 6.4 minutes (52 to 98)
b) Blood loss	80 + 10.4ml (62 to 102)
c) C-arm exposure	48 + 8 (36 to 81)
Union time	10.2 + 0.98 weeks (9 to 14)
Alignment at final outcome	
a) Coronal alignment	2.5° + 0.38° (1.8° to 2.9°)
b) Sagittal alignment	5.1° + 0.42° (3.4° to 5.6°)
Alignment immediate post-operatively	
a) Coronal alignment	3.2° + 0.47° (1.6° to 3.9°)
b) Sagittal alignment	6.83° + 0.51° (4.9° to 7.1°)
AOFAS Score	86.3 + 6.2 (70 to 93)

Mean duration of surgery, blood loss and C arm exposure were 79 minutes (range 52 to 98 min), 80ml (range 62 to 102ml) and 48 shoots (range 36 to 81 shoots), respectively.

**Fig. 2:**
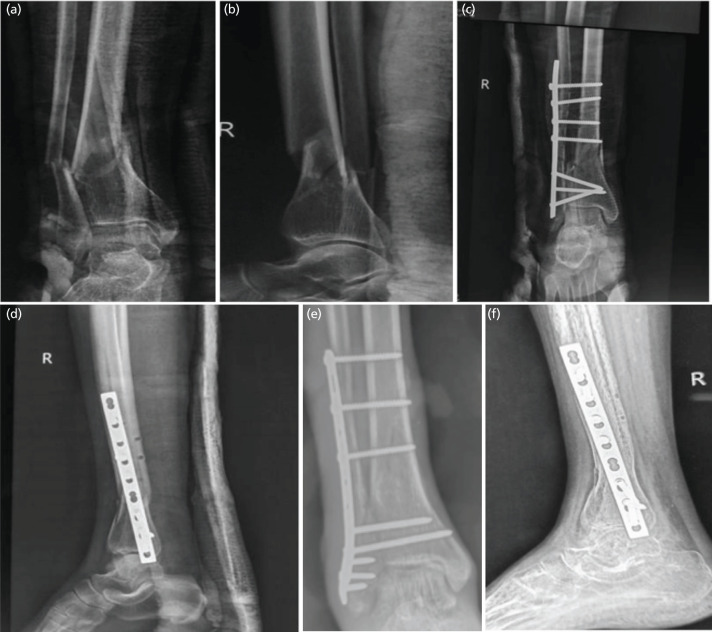
Radiographs of 30-year-old patient with distal tibial fracture managed with fibula pro tibia, (a, b) pre-operative, (c, d) immediate post-op, and (e, f) after one year.

**Fig. 3:**
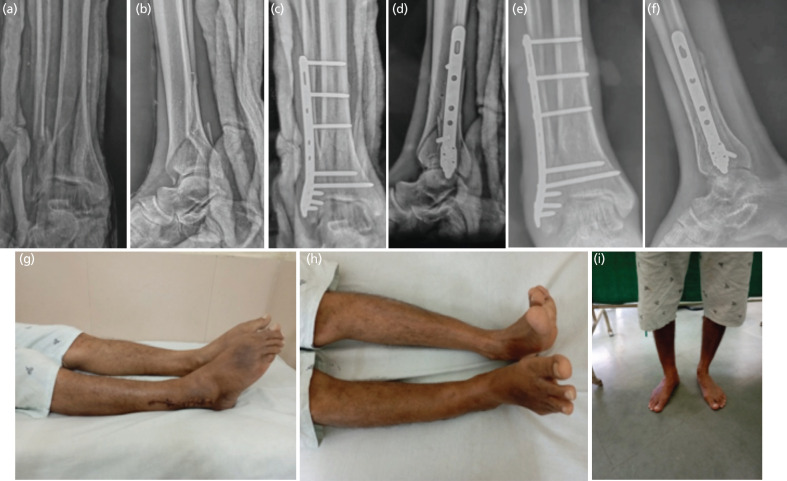
(a, b) AP and lateral pre-operative, (c, d) immediate, and (e, f) six-month follow-up radiographs. (g, h, i) Clinical pictures of a 26-year-old male with right side distal tibial fracture managed with fibula pro tibia plating, showing good function, alignment, and union.

Union occurred in all the patients without any need for augmentation. The mean union time was 10.2 weeks (range 9 to 14 weeks). Acceptable alignment was obtained in all the patients except one with mean coronal plane and sagittal alignment of leg improved from 11.67° and 13.69° pre-operatively, to mean 2.5° and 5.1° immediate postoperatively and 3.2° and 6.83° at one year follow-up, respectively (Fig. 2 and 3).

Mean AOFAS score in the series was 86.3 (range 70 to 93), with 20 cases having excellent score and 9 cases of good score and one case with fair result. None of cases had poor results. Mean ankle range of motion was 30° or more in 26 patients (normal or mild restriction) and four patients had 15° to 29° range having moderate restriction and only one of patient had severe restriction (<15°). None of the patients had any limitation to the daily activities and only two patients had limitation of recreational activities.

None of the patient had skin necrosis, wound dehiscence or healing problems, gait abnormality, non-union or implant failure, breakage or impingement. In one patient who had open fibular fracture of grade 3A, late infection occurred along the fibular plate side, which healed after implant removal. In one patient varus malunion was seen, as in this case tibial fracture was severely comminuted.

## Discussion

Distal tibial fractures with or without involvement of articular margin, account for 10 to 20% of all tibial fractures and 3 to 7% of all fractures. Ninety percent of these have associated fibular fracture and 12 to 55% are open fractures^1-4^. Peculiar anatomy, poor surrounding soft tissue sleeve, tenuous blood flow, high energy trauma, complex and compound fracture pattern and limited fixation options, leads to high complication rates ranging from 20 to 50 %^5-7^. Main reason behind this unacceptable high complication rate despite best surgical procedure is due to, severe iatrogenic trauma caused during open reduction or aggressive surgical technique in an already damaged soft tissue by initial trauma, in naturally susceptible area of tibia due to subcutaneous nature and tenuous blood supply^14^.

There is no uniform universally accepted treatment available for these fractures and options range from nonsurgical to surgical options of external fixation, limited open reduction, open reduction and internal fixation to primary arthrodesis, all having their own advantages and disadvantages^5-7^. Although the status of the soft tissue guides the exact treatment, the principle of surgical fixation remains the same, which includes management soft tissue and its gentle handling, anatomically reduced joint surface, restoration of limb alignment, stable construct and provision for early rehabilitation and mobilisation^11^.

Sequential four crucial steps in the management of these fractures as given by Reudi and Allgower are - primarily reduction and fixation of the fibula, followed by restoration of the tibial articular surface to maintain joint congruity, then filling of the metaphyseal bone defects caused due to depressed fractures by bone grating and finally, fixation of the metaphysis to the diaphysis with medial plate^15^.

Thus, reduction and fixation of fibula is important first step in management of these fractures. Fibular reduction and fixation help to maintain proper length, rotation and alignment and facilitates indirect tibial reduction of the anteriolateral (tillaux-chaput) and posteriolateral (volkmans) fragments through ligamentotaxis. It also provides a reference for reconstruction of distal tibia and prevents valgus malreduction^16-18^. Restoring the length by internal fixation of the fibula, has been mounted similar to temporary ankle spanning external fixator, which can be helpful in later realignment in same way as external fixators during two staged protocol^18,19^.

The fibular plate, which fixes the fibular fracture and facilitated indirect reduction of the tibial fracture, can also be used to fix the tibial fracture simultaneously, maintaining the reduction, alignment and rotation. This fixation of both fibula and tibia using a single plate is done by passing fibula pro tibia screws, which are longer screws passed through the plate fixing both the bone together. Fibula pro tibia, is a concept which relies on fact that fibular diaphysis, though is much smaller than the tibia, is very strong cortical bone. It holds strength for a longer duration, even with weight bearing, allowing good bone purchase. This fibula-tibial plate construct creates a stable frame which enhances healing^20-22^. Since the plate is applied via lateral approach exposing the fibula only, the soft tissue sleeve over the tibia is not disturbed at all. This does not cause any iatrogenic damage to the already compromised medial soft tissue, which is the main reason for delayed union and wound healing problems with open reduction.

There is very limited literature on fibula pro tibia plating, more so over none of them has been done for fresh distal tibial fractures. Said *et al* and DeOrio *et al* successfully used this concept of fibula pro tibia plating in tibial and peri-plafond tibial non-unions, respectively^20,21^.

Panchbhavi *et al* passed tibia pro fibula screws across the syndesmosis for osteoporotic bimalleolus fractures, even with intact syndesmosis and on comparing it with standard fibular plate fixation, found satisfactory alignment and union in both the groups but reduced complication rates of wound break down, infection, delayed healing and malunion in tibia pro fibula group^22^. Some authors have also used same technique to improve the stability in bimalleolus fractures in diabetics with good results^23-25^.

We used this fibula pro tibia plating technique in management of 30 fresh distal tibial and fibula fractures with mean age of 39.4 years and mean follow-up of 14.4 months and evaluated the outcome. Limited internal fixation was done for intra-articular fractures using k wire or screws if needed. We found mean AFOAS score in our study of 86 with almost 29 patients out of 30 patients having good to excellent results. Except for one case of infection and one case of varus malunion, we did not encounter any wound breakdown or healing problems or non-union. In our series, mean union time was 10.4 weeks, which seems to occur earlier than other reported series.

Fibula pro tibia plating as applied via lateral approach on fibula, stabilises lateral pillar and leaves the medial tibial soft tissue sleeve and periosteum intact. No tibial incision or medial exposure and not applying any type of plate on tibial surface, will prevent the surgical injury to the tenuous tibial blood supply, skin and soft tissue surrounding the tibia medially. This intact periosteum and blood supply to the tibia will help in early union and will avoid any wound related complications. Fibular cortical strut, locking fibular plate and fibula pro tibia screws with each screw having four cortical hold provides very high strength to the construct which can very well hold the tibial fracture fragments in reduced and aligned position. Further application of just one plate laterally on fibula in comparison to two plates of both fibula and tibia, will also decrease the surgical time, blood loss, radiation exposure, infection rate and cost. With fibula pro tibia plating there is risk of limitation of ankle range of motion and screw breakage like syndesmotic screws failure occurs if not removed. Since more than one fibula pro tibia screws are placed, the stresses on the individual screws are dissipated and prevent breakage. Placing the screw with ankle in maximal dorsiflexion prevents the narrowing of ankle mortise and hence preserve ankle mobility.

Our study is limited by lack of comparison groups and longer follow-ups. Further the study may also have selection bias, with inclusion of both extra-articular and intra-articular fractures along with open fractures in the study. Since this is a pilot study, further research with higher level of evidence, long term follow-up and comparison with other modes of treatment needs further research.

## Conclusion

Fibular plating with extended longer fibula pro tibia screws fixing fibula and tibia together, can be successfully used to manage complex distal tibial fractures. It is advantageous as it leaves the medial soft tissue and periosteal sleeve undisturbed, thus avoiding wound related problems.
